# Comprehensive Aerodynamic and Physicochemical Stability Evaluations of Nanocrystal-Based Dry Powder Inhalers: The Role of Mannitol and Leucine in Enhancing Performance

**DOI:** 10.3390/pharmaceutics17040436

**Published:** 2025-03-28

**Authors:** Heba Banat, Attila Nagy, Árpád Farkas, Rita Ambrus, Ildikó Csóka

**Affiliations:** 1 Institute of Pharmaceutical Technology and Regulatory Affairs, Faculty of Pharmacy, University of Szeged, Eötvös Street 6, 6720 Szeged, Hungary; banat.habosh@gmail.com (H.B.); csoka.ildiko@szte.hu (I.C.); 2 HUN_REN Wigner Research Centre for Physics, Konkoly Thege Miklós Street 29-33, 1121 Budapest, Hungary; nagy.attila@wigner.hun-ren.hu; 3 Institute for Energy Security and Environmental Safety, HUN-REN Centre for Energy Research, Konkoly Thege Miklós Street 29-33, 1121 Budapest, Hungary; farkas.arpad@ek-cer.hu

**Keywords:** nanotechnology, nanocrystal, dry powder, nano spray drying, mannitol, leucine, aerodynamic characteristics, stability

## Abstract

**Background**: Nanocrystals, a carrier-free nanotechnology, offer significant advantages for pulmonary drug delivery by enhancing the dissolution and solubility of poorly soluble drugs while maintaining favorable biological properties and low toxicity. This study aims to investigate the aerodynamic performance and stability of nanocrystal-based dry powders (NC-DPs). **Methods**: Nanocrystalline suspensions were produced via wet media milling and subjected to stability studies before undergoing nano spray drying. A factorial design was employed to optimize the process parameters. The influence of mannitol and leucine, individually and in combination, was evaluated in terms of aerodynamic properties (Aerodynamic Particle Sizer (APS), in silico modeling) and the physicochemical stability at room temperature (in a desiccator) and accelerated conditions (40 ± 2 °C, 75 ± 5% relative humidity). **Results**: APS analysis revealed that leucine-containing powders (K-NC-Ls) exhibited the smallest median (1.357 µm) and geometric mean (1.335 µm) particle sizes, enhancing dispersibility. However, in silico results indicated the highest exhaled fraction for K-NC-L, highlighting the need for optimized excipient selection. Although mannitol showed the lowest exhaled fraction, it was mainly deposited in the extra-thoracic region in silico. The mannitol/leucine combination (K-NC-ML) revealed a low exhaled fraction and high lung deposition in silico. Also, K-NC-ML demonstrated superior stability, with a 6% reduction in D[0.5] and a 5% decrease in span overtime. Furthermore, no significant changes in crystallinity, thermal behavior, drug release, or mass median aerodynamic diameter were observed under stress conditions. **Conclusions**: These findings confirm that combined incorporation of mannitol and leucine in NC-DP formulations enhances stability and aerodynamic performance, making it a promising approach for pulmonary drug delivery.

## 1. Introduction

Nanotechnology offers significant advantages in pulmonary drug delivery, particularly for poorly soluble drugs, by overcoming biological and physical barriers, improving solubility, and enhancing bioavailability [1,2]. As absorption is more efficient, lower doses can be used, thereby reducing the risk of adverse effects. Nanoparticles used in pulmonary delivery can be categorized into carrier-based and carrier-free types according to their composition and properties. Lipid-based nanoparticles, such as liposomes; solid lipid nanoparticles; and nanoemulsions are widely used for their ability to encapsulate both hydrophilic and hydrophobic drugs [3,4]. Polymeric nanoparticles, including PLGA and chitosan, are valued for their biodegradability, biocompatibility, and mucoadhesive properties, which enhance drug retention in the lungs [5,6,7,8]. Inorganic nanoparticles, like mesoporous silica, offer high surface area and high loading capability, suitable biocompatibility, and their surface can be functionalized [9]. Hybrid nanoparticles, which combine materials such as lipids and polymers, are designed to optimize drug delivery by leveraging the advantages of multiple components [10]. Each type of nanoparticle offers unique benefits, making them suitable for various therapeutic and diagnostic applications in pulmonary delivery.

Nanocrystals, a carrier-free nanotechnology, have gained increasing attention for pulmonary administration due to their ability to enhance the dissolution rate and saturation solubility of poorly soluble drugs while maintaining favorable biological properties and low toxicity [11]. Drug nanocrystals are typically pure drug particles ranging from 1 to 1000 nm in size and are stabilized by surfactants or stabilizers without requiring additional carrier materials [12]. This technology enhances drug adhesion to biological membranes, increases saturation solubility, and improves bioavailability due to its high surface area. Nanocrystal formulations have been explored for various administration routes, including oral, intravenous, pulmonary, percutaneous, and ocular delivery. In pulmonary drug delivery, nanocrystals offer unique advantages, such as high drug loading, improved mucus penetration, reduced macrophage clearance, and enhanced drug release, making them a promising option for inhalation administration [13].

The top–down approach is commonly used for nanocrystal preparation, where larger solid particles are mechanically reduced to nanoscale sizes. This method includes techniques such as media milling, high-pressure homogenization, and microfluidization. Among these, wet media milling is widely employed due to its simplicity, reproducibility, and eco-friendliness, as it does not require organic solvents [14]. Wet media milling is an established pharmaceutical technique for producing stable nanosuspensions of poorly water-soluble active pharmaceutical ingredients (APIs), with scalability further enhanced by modeling techniques to optimize industrial applications and improve efficiency [15]. However, to obtain an inhalable dry powder, an additional solidification step is required.

Spray drying is a widely used technique for converting liquid nanosuspensions into dry powders with improved stability and ease of administration [16]. Compared to conventional spray drying, nano spray drying offers superior control over droplet formation, resulting in more uniform particles with narrow size distribution. Unlike traditional spray dryers, which rely on pressure or centrifugal force, nano spray dryers use a vibrational nebulizer to generate uniform microdroplets, leading to higher yields even from small sample quantities [17,18]. Despite these advantages, nano spray drying has been underutilized in developing DPIs compared to conventional spray dryers, presenting an opportunity for improvement. The combination of wet media milling and spray drying has recently gained interest for developing nanocrystal-based dry powder inhaler (DPI) formulations.

The selection of excipients during spray drying significantly influences the physicochemical properties of the final powder. Common excipients, such as mannitol, trehalose, and leucine, play a critical role in stabilizing formulations during drying and ensuring product stability. Mannitol is commonly used as a cryoprotectant, preserving structural integrity and enhancing formulation stability [19]. However, its tendency to crystallize can impact the aerosol performance and fine particle fraction (FPF) upon storage [20]. In contrast, leucine improves powder dispersibility and stability in dry powder formulations by reducing particle cohesion and enhancing aerosolization [21]. Since excipients in spray drying serve multiple roles, including thermal protection, aggregation prevention, and maintaining API bioactivity, the ideal spray-dried preparations may necessitate a combination of various excipients [22]. Moreover, their influence on spray-dried powder should be carefully evaluated to ensure optimal characteristics and to achieve the desired powder properties.

Aerodynamic analysis plays a crucial role in DPI formulation, as the effectiveness of inhaled drugs is directly influenced by the aerodynamic properties of aerosolized particles [23]. The aerodynamic diameter of particles determines their deposition in the respiratory tract, with optimal pulmonary delivery occurring within the 1–5 µm range. Particles within this range efficiently reach the alveolar region, ensuring effective treatment of respiratory diseases [24]. Various factors, including particle size distribution, shape, density, and flow properties, influence aerodynamic behavior [25]. A comprehensive evaluation of these characteristics is essential to confirm the suitability of a formulation for inhalation and therapeutic efficacy.

Stability is another key consideration in DPI formulation, as storage circumstances can significantly affect particle shape and aerosol performance [26]. While solid formulations largely offer better stability than liquid systems, nanosized APIs are prone to aggregation, which can compromise functionality [14]. Crystalline APIs are often preferred over amorphous forms due to their superior physical stability, though amorphous APIs can provide improved dissolution and bioavailability [27,28]. However, the influence of storage conditions—particularly relative humidity—on stability is highly drug specific [29]. Despite extensive research on the stability of dry powder formulations [20,28,30,31,32,33], stability assessments remain essential for every newly developed DPI. Ensuring a balance between stability and performance is crucial to mitigating challenges such as crystal growth, sedimentation, and agglomeration, ultimately safeguarding the efficacy and safety of the final product.

Thus, the present study aims to evaluate the aerodynamic performance and stability of nanocrystal-based DPI formulations (NC-DPs). The nanocrystalline suspension produced via wet media milling was subjected to stability studies before undergoing nano spray drying. Box–Behnken factorial design (BBD) was employed to optimize the process parameters. The effects of excipients, including mannitol and leucine (used individually or in combination), were assessed on particle size, aerodynamic characteristics, and physicochemical stability under normal storage conditions (desiccator) and accelerated stress conditions (40 ± 2 °C and 75 ± 5% relative humadity (RH)), following International Council for Harmonisation of Technical Requirements for Pharmaceuticals for Human Use (ICH) guidelines. Aerodynamic properties were analyzed using an Aerodynamic Particle Sizer (APS) and in silico modeling. Furthermore, the physicochemical stability of the formulations was examined using laser diffraction, scanning electron microscopy (SEM), differential scanning calorimetry (DSC), X-ray powder diffraction (XRPD), dissolution testing, and Andersen cascade impaction. By addressing these critical factors, this study aims to validate the formulation’s suitability for pulmonary drug delivery and assess its feasibility for future clinical applications.

## 2. Materials and Methods

### 2.1. Materials

Ketoprofen (KTP) (TCI Chemicals, Shanghai, China) was used as the active pharmaceutical ingredient (API) in this study. Polyvinyl alcohol (PVA) (ISP Customer Service GmbH, Cologne, Germany), a biocompatible and non-toxic polymer, and sodium dodecyl sulfate (SDS) (VWR Chemicals, Leuven, Belgium), an anionic surfactant, were employed as stabilizing agents. D-mannitol (Molar Chemicals Ltd., Budapest, Hungary) and L-leucine (AppliChem GmbH, Darmstadt, Germany) were incorporated during the nano spray drying process. Distilled water used in the experiments was obtained from a Milli-Q system (Millipore, Merck KGaA, Darmstadt, Germany).

### 2.2. Nanocrystal Feed Preparation

A KTP-containing nanosuspension (KTP-NS) was formulated based on a previously reported method, with a slight modification [34]. In brief, drug particles were dispersed in an aqueous solution containing PVA (1% *w*/*v*) and SDS (0.1% *w*/*v*). The combination of steric (PVA) and electrostatic (SDS) stabilizers helps maintain nanocrystal stability [35]. Then, wet media milling was performed in a planetary ball mill (Retsch Planetary Ball Mill PM 100 MA, Retsch GmbH, Haan, Germany) using 20.00 g of 0.3 mm zirconium dioxide (ZrO_2_) beads. The milling process was carried out at 400 rpm for 90 min in a 50 mL milling chamber, with a pause every 15 min for 5 min to allow cooling.

### 2.3. Inhalable Nanocrystal-Based Dry Powder (NC-DP) Preparation

#### 2.3.1. Optimization of Nano Spray Dryer Parameters by Box–Behnken Factorial Design (BBD)

A Büchi Nano Spray Dryer equipped with a medium nebulizer (Büchi Nano Spray Dryer B-90 HP, Büchi, Flawil, Switzerland) was used to produce the NC-DPs. The Box–Behnken factorial design (BBD) was employed to optimize the parameters of the nano spray dryer, assisted by TIBCO Statistica^®^ 13.4 (Statsoft Hungary, Budapest, Hungary) for experimental design generation. Three independent factors were chosen: pump (%), temperature (°C), and feed concentration (% *w/v*). These factors play crucial roles in the nano spray drying process. Pump dictates the droplet size during atomization [36], thus affecting the size and uniformity of the resulting NC-DP. Temperature influences drying rate and solid-state properties, while feed concentration impacts both evaporation dynamics and yield percentage [37]. The effect of these factors on the dependent variables (D[0.5], span, and yield percentage) was investigated at three levels, as outlined in Table 1.

Temperature was studied at 90, 100, and 110 °C, considering the melting point of KTP is 94 °C [38], and taking into account the boiling point of water, which is appropriate for the water-based formulation. Feed concentration was selected at the highest concentration achievable from the media milling process (10% *w*/*v*) [39] and then further diluted to 5% and 2.5% (*w*/*v*). The pump percentages were chosen to explore a wide range of feed rates: 10% to examine slow feed rates and finer particles, 30% for an intermediate balance between droplet formation and drying efficiency, and 50% to investigate the effects of high feed rates on particle formation and yield [36]. Moreover, mathematical models were developed for each dependent variable, and the relationship of the variables in the response was analyzed by the following second-order polynomial equation:

(1) $$y=\beta_{0}+\beta_{1}X_{1}+\beta_{2}X_{2}+\beta_{3}X_{3}+\beta_{12}X_{1}X_{2}+\beta_{13}X_{1}X_{3}+\beta_{23}X_{2}X_{3}+\beta_{11}X_{1}^{2}+\beta_{22}X_{2}^{2}+\beta_{33}X_{3}^{2}$$

where y is the response variable; β_0_ is a constant; β_1_, β_2_, and β_3_ are linear coefficients; β_11_, β_22_, and β_33_ are quadratic coefficients; β_12_, β_13_, and β_23_ are the interaction coefficients; X_1–3_ are the main effect factors; X_12_, X_22_, and X_32_ are the quadratic effect factors; and X_1_X_2_, X_1_X_3_, and X_2_X_3_ are the interaction effect factors. An analysis of variance (ANOVA) was conducted, with significance determined at a p-value less than 0.05, indicating statistical significance at the 95% confidence level. Response surface plots, in the form of 3D surface plots, were generated for D[0.5] (µm), span, and yield (%) based on the regression model, with one variable held constant at its center level.

#### 2.3.2. Mannitol/Leucine Incorporation

The optimized parameters obtained from the BBD were utilized to extensively investigate the effects of leucine and mannitol, two widely employed excipients in inhaled preparations, on the properties of the NC-DP. Both mannitol and leucine are known to enhance re-dispersibility upon hydration, improve the drug dissolution, and boost aerosol performance [40]. Using optimized nano spray dryer parameters, we evaluated the impact of excipients—mannitol (K-NC-M), leucine (K-NC-L), their combination (K-NC-ML), and the absence of excipients (K-NC)—on the particle size, aerosolization properties, and stability of NC-DPs for pulmonary delivery. In this context, a magnetic stirrer (AREC. X heating magnetic stirrer, Velp Scientifica Srl, Usmate Velate, Italy) was used for dissolving mannitol and/or leucine in the nanosuspension. Based on the factorial design study, the formulations were prepared as detailed in Table 2; mannitol and/or leucine were added accordingly while ensuring a constant total concentration.

### 2.4. Stability of the Nanosuspension

To assess the stability of the KTP-NS prior to the nano spray drying process, a short-term stability study was conducted. The nanosuspension was stored at refrigeration temperature (+4 °C) and evaluated for particle size and distribution at predefined time intervals using the NanoSight NS 3000 system (Malvern Instruments, Worcestershire, UK). Nanoparticle Tracking Analysis (NTA) was employed for precise particle size measurements. The instrument featured a 565 nm laser, a high-sensitivity sCMOS camera, and a syringe pump. The refractive index for KTP was set at 1.592, and the nanosuspension was diluted before being analyzed. The diluted sample was introduced into the instrument at a syringe pump speed of 50. Measurements were taken by recording three 30-s videos in script control mode, producing a total of 1500 frames for analysis.

The particle size distribution was characterized using the parameters D[0.1] (indicating the size below which 10% of the volume distribution falls), D[0.5] (the median particle size), and D[0.9] (the size below which 90% of the volume distribution falls). Additionally, the size distribution span was calculated using Equation (2). After confirming the stability of the NS, the nano spray dryer was employed (as detailed in Section 2.3), and powder characterizations were conducted.

(2) $$Span=\frac{D\left[0.9\right]-D\left[0.1\right]}{D\left[0.5\right]}$$

### 2.5. Particle Size and Yield

Laser diffraction was used to assess the particle size and distribution of the nano spray-dried NC-DPs with a Malvern Mastersizer Scirocco 2000 device (Malvern Instruments Ltd., Malvern, UK). A dry dispersion unit was employed with 0.5–1.0 g of powder placed in the feeding tray. The refractive index for KTP was set at 1.592, and the dispersion air pressure was adjusted to 3.0 bar with a vibration feed of 75%. Triplicate measurements were performed for each sample. The particle size distribution was reported using D[0.1], D[0.5], and D[0.9] values, and the span was calculated using the previously defined equation (Equation (2)). The mass ratio of dry powder collected after spray drying to the initial solid compositions before drying was calculated to determine the percentage yield of each sample (Equation (3)).

(3) $$Yield\left(\%\right)=\frac{Weight\;of\;dry\;powder\;collected\left(after\;spray\;drying\right)}{Weight\;of\;the\;initial\;solid\;composition\left(before\;spray\;drying\right)}\times 100\%$$

### 2.6. Aerosol Performance via Aerodynamic Particle Sizer

The powders were filled into size 3 Ezeeflo™ hydroxypropyl methylcellulose (HPMC) capsules (ACG-Associated Capsules Pvt. Ltd., Mumbai, India) and tested using the Breezhaler^®^ dry powder inhaler DPI (Novartis International AG, Basel, Switzerland). The experimental setup included an induction port simulating the upper respiratory tract, a high-capacity vacuum pump (HCP5; Copley Scientific Ltd., Nottingham, UK) with a critical flow controller (TPK 2000; Copley Scientific Ltd., Nottingham, UK), and an Aerodynamic Particle Sizer (APS) (TSI Incorporated, Shoreview, MN, USA).

To simulate inhalation, the vacuum pump activated the DPI with a rectangular 4-s breathing waveform at a flow rate of 60 L/min. The APS sampled particles from the airflow via an isokinetic nozzle, using a TSI 3321 model for size distribution analysis. This instrument measures aerosol particle size distributions with aerodynamic diameters ranging from 0.5 to 20 μm across 52 channels. It determines particle sizes by measuring their time-of-flight within an accelerated flow.

The APS sampling flow rate was set at 1 L/min, with a 5-s sampling duration and no pauses. This duration was chosen based on the length of the inhalation profile and the particles’ residence time in the measurement system. A TSI 4000 thermal mass flow meter (TSI Incorporated, Shoreview, MN, USA), with a measuring range of 0.5–200 NL/min, was used to regularly verify the 60 L/min flow rate during measurements.

### 2.7. In Silico Aerodynamic Analysis

The latest version of the Stochastic Lung Model (SLM) was utilized to estimate drug deposition across different anatomical regions of the airways, expressed as regional deposition fractions [41]. Numerical modeling offers several advantages over traditional scintigraphic studies, which often require large populations and face significant technical and ethical challenges [42]. This non-invasive, reproducible, and efficient method has proven to be a valuable tool for quantifying the distribution of medications in various parts of the respiratory tract, including total, local, and regional depositions.

In this study, we employed a validated airway deposition model to numerically calculate the deposition fractions in the lungs and upper airways (extra-thoracic region). Deposition fraction refers to the proportion of the drug mass deposited in a specific airway region relative to the total drug mass loaded into the capsule. Lung deposition fraction is determined as the sum of the bronchial and acinar deposition fractions. Meanwhile, the exhaled fraction is calculated by subtracting the total deposited fractions (lungs, upper airways, and remaining in the device) from the metered fraction (100%).

The model incorporates a Monte Carlo approach, which uses stochastic simulations to account for the natural variability in airway morphology. By tracking approximately 10⁵ particles from inhalation to exhalation, the model can simulate their deposition in various lung regions or their exhalation. Key inputs for the model include particle characteristics and inhalation parameters. For this study, the inhalation parameters were tailored to match those of patients with chronic obstructive pulmonary disease (COPD), with a peak inhalation flow of 69.5 L/min, an inhaled volume of 1.7 L, and an inhalation time of 2.04 s [25].

Additionally, the study investigated how breath-hold (BH) duration impacts deposition patterns by applying BH times of 5 and 10 s. The numerical deposition model used here was previously validated specifically for aerosolized medications [43], ensuring its reliability for this application.

### 2.8. Long-Term Stability Under Normal Conditions

To assess the long-term stability of the NC-DPs, the four samples were stored in opaque, well-sealed glass containers at room temperature in a desiccator for six months. The particle size was evaluated using laser diffraction analysis (as described in Section 2.5). The particle size was characterized using D[0.1], D[0.5], D[0.9], and span. The percentage change in particle size after six months of storage was calculated relative to the initial values (0 months, before storage) to quantify any changes over time (Equation (4)).

(4) $$Change(\%)=\frac{Value\;at\;6\;months-Value\;at\;0\;months}{Value\;at\;0\;months}\times 100\%$$

### 2.9. Stability Under Accelerated Conditions

Stability testing was performed in a Binder KBF 240 environmental chamber (Binder GmbH, Tuttlingen, Germany), featuring an APT.line™ preheating and cooling system for precise temperature control. The chamber maintained consistent conditions across a temperature range of 10–70 °C and RH levels of 10–80%, ensuring reproducibility throughout the study. In accordance with ICH guidelines, the stability test was carried out at 40 ± 2 °C and 75 ± 5% RH. The formulations were placed in size 3 Ezeeflo™ hydroxypropyl methylcellulose capsules (ACG-Associated Capsules Pvt. Ltd., Mumbai, India) and subjected to a 90-day stability assessment. Analytical samples were collected at three time points: initial (time zero), intermediate (30 days), and final (90 days) storage intervals.

#### 2.9.1. Morphology

The shape of both freshly prepared and stored NC-DPs samples was examined using a scanning electron microscope (SEM) (Hitachi Scientific Ltd., Tokyo, Japan). The SEM was operated at an accelerating voltage of 10 kV, and the samples were coated with a thin gold–palladium layer using a Bio-Rad sputter coater (2.0 kV, 10 mA, for 10 min). The coating process was conducted under an air pressure range of 1.3–13.0 mPa.

#### 2.9.2. Thermal and Solid-State Analysis

Differential scanning calorimetry (DSC) was carried out by operating a Mettler Toledo DSC 821e system, controlled by STARe thermal analysis software version 9.3 (Mettler Inc., Schwerzenbach, Switzerland). For the analysis, approximately 2–3 mg of each sample and physical mixture (corresponding to sample compositions) was sealed in hermetically closed aluminum pans with pierced lids, while an empty pan served as the reference. The temperature was scanned from 25 °C to 300 °C at a heating rate of 10 °C/min, using argon as the carrier gas with a flow rate of 10 L/h.

The samples and KTP raw were investigated using a BRUKER D8 Advance X-ray powder diffractometer (Bruker AXS GmbH, Karlsruhe, Germany) for structural analysis. The system utilized a Cu Kα1 radiation source (λ = 1.5406 Å) and a VÅNTEC-1 detector. Scans were performed with a copper target and nickel filter, operating at 40 kV and 40 mA. The measurements covered a 2θ range of 3° to 40°, with a step size of 0.01° and a step time of 0.1 s.

#### 2.9.3. Aerodynamic Characterization by Andersen Cascade Impactor

The aerosolization behavior of fresh prepared and stored NC-DP samples was evaluated using an Andersen Cascade Impactor (ACI) (Copley Scientific Ltd., Nottingham, UK). An inhalation flow rate of 60 L/min was maintained using a High-Capacity Pump (HCP5), along with a Critical Flow Controller (TPK, Copley Scientific Ltd.), with the flow rate confirmed by a mass flow meter (DFM 2000, Copley Scientific Ltd.). The samples were placed in size 3 Ezeeflo™ HPMC capsules (ACG-Associated Capsules Pvt. Ltd., Mumbai, India) and administered via a Breezhaler^®^ single-dose inhaler (Novartis International AG, Basel, Switzerland). Upon activation, aerosolization was carried out over a 4-s inhalation phase. To mimic lung adhesion, the ACI plates were pre-treated with a 1:99 (*v*/*v*%) mixture of Span 85 and cyclohexane, which was allowed to dry prior to testing. After aerosol deposition, each ACI component was washed with 50% methanol (*v*/*v*) to recover the deposited KTP. UV/VIS spectrophotometry (ATI-UNICAM UV/VIS Spectrophotometer, Cambridge, UK) was employed to measure KTP concentrations at 258 nm. The in vitro aerodynamic properties were analyzed using Inhalytix™ software (Copley Scientific Ltd., Nottingham, UK; Available online: https://www.copleyscientific.com/inhaler-testing/apsd-data-analysis-software/inhalytix/, accessed on 10 October 2024) with key performance metrics calculated as follows:

- MMAD (Mass Median Aerodynamic Diameter): represents the effective particle size during inhalation.
- FPF (Fine Particle Fraction): indicates the percentage of drug particles smaller than 5 µm.
- EF (Emitted Fraction): denotes the percentage of drug mass reaching the impactor relative to the initial amount of KTP loaded.

#### 2.9.4. Dissolution Test in Simulated Lung Media

A modified paddle method (Hanson SR8 Plus, Teledyne Hanson Research, Chatsworth, CA, USA), adhering to European Pharmacopeia guidelines, was used to evaluate KTP release. To accommodate the study, 100 mL vessels and smaller paddles were substituted for the standard 1000 mL vessels. Simulated lung fluid (SLF) was prepared to mimic human airway conditions, consisting of sodium chloride (NaCl), sodium bicarbonate (NaHCO_3_), calcium chloride (CaCl_2)_, sodium dihydrogen phosphate (NaH_2_PO_4_), sulfuric acid (H_2_SO_4_), and glycine, adjusted to a pH of 7.4. Each vessel was filled with 50 mL of SLF and maintained at 37 °C. The NC-DP samples, each containing 5 mg of KTP, were dispersed in the SLF, and the paddle was set to rotate at 50 rpm for 60 min. Samples of 5 mL were collected at intervals of 5, 10, 15, 30, and 60 min and immediately replaced with fresh SLF to maintain sink conditions. Collected samples were filtered using a Millex-HV syringe-driven filter unit with a 0.45 µm pore size (Millipore Corporation, Bedford, MA, USA) and analyzed via UV/VIS spectrophotometry at 258 nm (ATI-UNICAM UV/VIS Spectrophotometer, Cambridge, UK). All measurements were conducted in triplicate for accuracy and reproducibility.

### 2.10. Statistical Analysis

Statistical analysis was conducted using GraphPad Prism version 8.0.1 (GraphPad Software, CA, USA). A two-way ANOVA test was employed to observe statistical significance, with significant values at *p* ≤0.05 (* *p* ≤ 0.05; ** *p* ≤ 0.01; *** *p* ≤ 0.001).

## 3. Results and Discussion

### 3.1. Stability of the Nanosuspension

**Figure 1:**
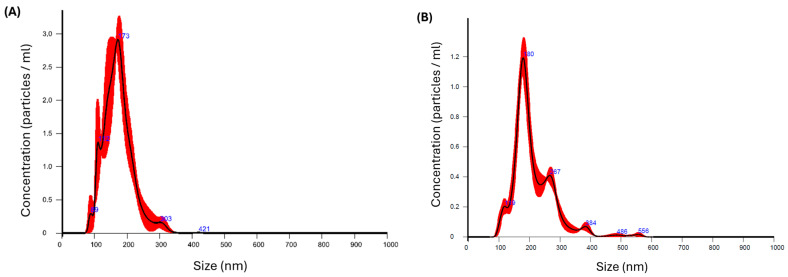
NTA results of the KTP-NS (**A**) as freshly prepared and (**B**) after 45 days storage at 4 °C (red color shows the particle distributions, and blue color shows the particle size at exact points). Data are means ± SD (*n* = 3 independent measurements).

The short-term physical stability of the KTP-NS was evaluated over 45 days at +4 °C (refrigerated) (Figure 1). After storage, particle size parameters, including D[0.1], D[0.5], D[0.9], and span, were analyzed. The D[0.5] exhibited a slight but significant increase of 29 nm (*p* ≤ 0.001); however, the span remained unchanged, indicating that the overall particle size distribution was preserved (Table 3).

The particle distribution (span) is particularly important for the nanosuspension stability, as it ensures consistent particle dispersion and prevents excessive aggregation, which is crucial for maintaining the formulation’s performance and stability over time [44]. In fact, selecting the right stabilizers is a crucial step in the wet media milling approach, often requiring significant effort and careful consideration [45]. Moreover, the stability of a nanosuspension primarily depends on the stabilizers used [46]. PVA enhances drug formulations by improving the wettability and dispersion of poorly soluble drugs. Its chemical stability ensures long-term formulation integrity, while its ability to form hydrogen bonds with active ingredients supports nanosuspension stability [47]. Notably, the use of sodium dodecyl sulfate (SDS) as an anionic surfactant performed a key role in maintaining the electrostatic stability of KTP-NS, thereby enhancing its overall stability. Several studies have demonstrated that combining stabilizers or surfactants can further improve nanosuspension stability [35,48,49]. Following successful completion of this stability test, the nanosuspension was deemed suitable for solidification and further processing into a dry powder.

### 3.2. Optimized Parameters of Nano Spray Dryer

BBD was implemented to optimize the process of nano spray drying by varying the parameters, i.e., pump (%) (X_1_), temperature (°C) (X_2_), and feed concentration (% *w*/*v*) (X_3_), in 15 experiments. The obtained results, i.e., particle size (D[0.5]), particle size distribution (span), and yield (%), are shown in Table 4.

**Figure 2:**
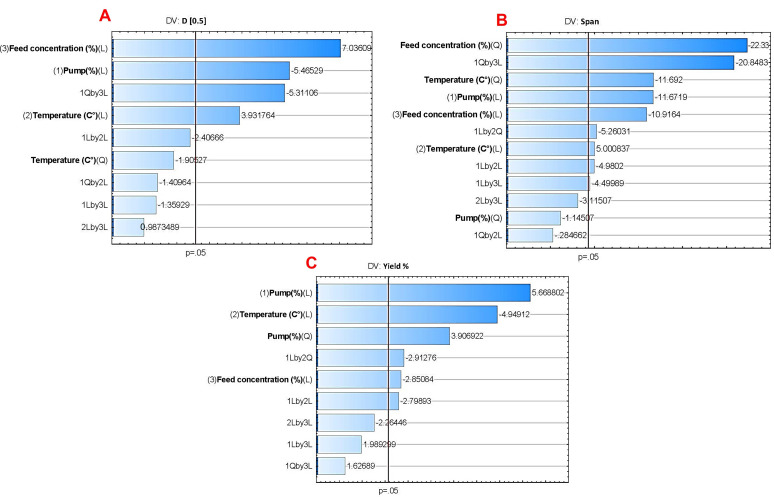
Pareto charts of the effect of the assessed independent variables on (**A**) D[0.5], (**B**) span, and (**C**) yield (%). Bars exceeding the vertical line indicate that the terms are significant (*p* < 0.05).

The particle size and particle size distribution are fundamental for determining the aerodynamic behavior in pulmonary delivery. While particle size (represented here by D[0.5]) affects the dispersibility, dissolution rate, drug release, and lung deposition efficiency of NC-DPs, span is crucial for ensuring a consistent particle size distribution and uniformity. A narrow span indicates uniform particle sizes, resulting in more effective aerosolization and better lung deposition. Figure 2A shows that D[0.5] was significantly influenced by all independent variables; however, feed concentration and pump (%) were the most significant. Span was found to be significantly affected by feed concentration, pump (%), and temperature (°C), as shown in Figure 2B. Yield (%) was mainly influenced by pump and temperature (Figure 2C). A high yield percentage ensures that the process is economically viable and sustainable, maximizing the utilization of resources [50]. Nevertheless, D[0.5] and span were prioritized for the optimization in this study.

After statistical analysis, second-order relationships for the estimation of D[0.5], span, and yield (%) were established, as explained through the following equations:

$$\begin{array}{c} D\left[0.5\right]=2.068772+0.144868x_{3}-0.112441x_{1}-{0.103857x}_{1}x_{3}^{2}+0.086722\;x_{2}-0.068250x_{1}x_{2}-\\ 0.037520x_{1}x_{3}-0.029042x_{1}x_{2}^{2}-0.028555x_{2}^{2}+0.028000x_{2}x_{3}\; \end{array}$$

$$\begin{array}{c} Span=50.8559-42.8158x_{1}x_{3}^{2}-34.6144x_{3}^{2}-{26.3660x}_{1}-23.6316x_{3}-18.1196x_{2}^{2}-\\ 14.4643\;x_{1}x_{2}-13.0692x_{1}x_{3}-11.0990x_{1}x_{2}^{2}+11.0648x_{2}-9.2369x_{2}x_{3}-1.7746x_{1}^{2} \end{array}$$

$$\begin{array}{c} Yield\;\left(\%\right)=64.7701+13.9411x_{1}-11.3528x_{2}-{8.8500x_{1}x}_{2}+6.9691x_{2}x_{3}-6.6908x_{1}x_{2}^{2}-\\ 6.5445x_{3}+6.5287x_{3}^{2}+6.2900x_{1}x_{3}+3.5471x_{1}x_{3}^{2} \end{array}$$

**Figure 3:**
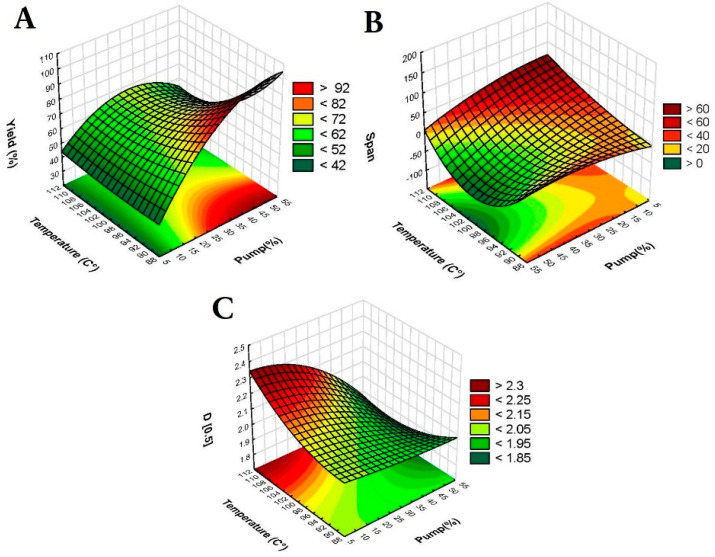
Three-dimensional surface plots of the effect of temperature (°C) and pump (%) on (**A**) yield (%), (**B**) span, and (**C**) D[0.5].

Additionally, the response 3D surface plots of temperature and pump and their effects on span, D[0.5], and yield are shown in Figure 3.

As the pump percentage increased, D[0.5] and span decreased, indicating enhanced atomization efficiency and finer droplet formation. Higher pump rates increase shear forces, resulting in smaller droplets that can dry quickly and uniformly. Furthermore, the increased feed rate enhances drying kinetics and optimizes the drying process, preventing droplet coalescence and resulting in smaller particles [51]. Conversely, it is known that higher feed concentrations lead to larger particle sizes after drying due to increased solute per droplet [52]. However, in our study, high span values were observed at low feed concentrations (2.5% *w*/*v*), possibly indicating less stable droplet formation and inconsistent solute distribution. Moreover, higher temperatures applied in this study (100 and 110 °C) potentially caused larger particles (higher D[0.5]) and broader particle size distributions (higher span values) due to rapid evaporation and possible uncontrolled aggregation [17]. Lower temperatures (90 °C) resulted in slower drying, leading to smaller, more uniform particles. Therefore, the parameters pump—50%, temperature—90 °C, and feed concentration—5% were selected, as they resulted in the lowest D[0.5] and span. Using these conditions, samples containing mannitol and/or leucine (as detailed in Table 2) were produced and evaluated for aerodynamic performance and stability.

### 3.3. Particle Size by Laser Diffraction

The results of laser diffraction are shown in Table 5. After process optimization of nano spray drying, the D[0.5] value of the optimized sample (K-NC) was 1.708 ± 0.074 µm. Leucine incorporation individually (K-NC-L) resulted in a slight increase in D[0.5] (1.893 ± 0.017 µm). Leucine, a surface-active agent, diffuses to the droplet surface during spray drying, stabilizing the droplet by creating a rigid shell, resulting in larger particles [53]. In contrast, mannitol in the K-NC-M sample significantly increased the particle sizes (3.337 ± 0.190 µm). Mannitol is known for its high tendency to crystallize during drying, promoting the formation of larger, well-defined particles [54]. However, the combined presence of both excipients (K-NC-ML) led to a moderate particle size (2.046 ± 0.064 µm), possibly due to a balance between leucine’s stabilizing effects and mannitol’s particle-enlarging effects. Nevertheless, the span values obtained with the presence of mannitol and leucine were lower than the sample without mannitol or leucine addition (K-NC), suggesting that these excipients contribute to a more uniform particle size distribution, enhancing aerodynamic properties for more uniform deposition throughout the respiratory tract.

### 3.4. Aerosol Performance

The Aerodynamic Particle Sizer (APS) was used to measure the aerodynamic diameter, particle size distribution, and number concentration—key parameters for predicting deposition in the respiratory tract [55]. Particles in the 1–5 µm range are optimal for deep lung deposition, while larger particles (>5 µm) tend to accumulate in the upper airways, reducing lung delivery efficiency [24]. To assess aerosol performance, three size-based parameters were analyzed: number particle size, surface particle size, and volume particle size. These metrics provide insights into particle aggregation, surface area distribution (which influences dissolution and bioavailability [56]), and mass distribution (crucial for dosing accuracy and therapeutic efficacy [57]).

**Figure 4:**
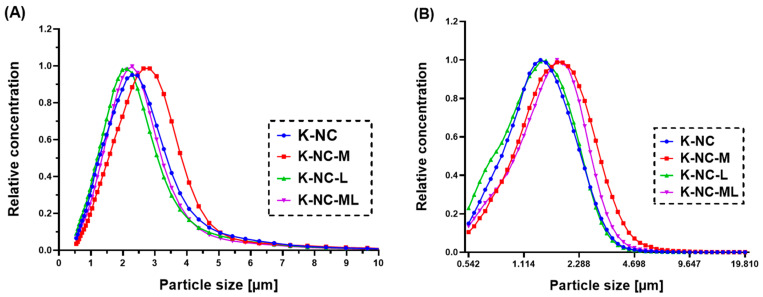
Results on Aerodynamic Particle Sizer (APS) for aerosol performance investigation of NC-DP samples’ (**A**) volume size distribution and (**B**) number particle size distribution. Data are means ± SD (*n* = 5 independent measurements).

Figure 4 presents the volume size and number particle size distributions of NC-DP samples. K-NC-M exhibited the broadest distribution with larger particle sizes (Figure 4A), which may hinder deep lung deposition. In contrast, K-NC-L and K-NC-ML demonstrated narrower, more favorable distributions, suggesting improved aerosol performance.

The number particle size data (Table 6) confirmed that K-NC-L (with leucine) had the smallest median (1.357 µm) and geometric mean (1.335 µm), while surface particle size analysis further highlighted its advantage, with K-NC-L showing the lowest median (2.394 µm) and geometric mean (2.692 µm). This aligns with leucine’s known role as a dispersibility enhancer, reducing aggregation and improving aerosolization [58,59]. Smaller, well-dispersed particles enhance dissolution and bioavailability, making leucine an effective stabilizer [60].

In contrast, K-NC-M (with mannitol) had larger particle sizes (median: 1.690 µm), indicating that mannitol alone may not sufficiently optimize the particle size for inhalation. Additionally, its larger surface area could negatively affect aerosol performance. The combination of mannitol and leucine in K-NC-ML resulted in intermediate particle sizes (median: 1.600 µm), offering a balance between the two excipients’ properties. This was in line with a previous study by Pasero et al. (2025), where the aerosolization properties microparticle system (mannitol and salbutamol sulphate) was significantly improved up to the maximum FPF (48%) and MMAD (2 μm) when combined and spray freeze-dried with leucine at 10% (*w*/*w* db) [61]. Another study found a highly significant and positive impact of leucine on enhancing the FPF of spray-dried mannitol particles [62].

Overall, while K-NC-M showed larger particle sizes and a broader distribution, K-NC-ML provided a more balanced aerosol performance. These findings underscore the importance of excipient selection in dry powder formulations, with leucine emerging as a key component for optimizing particle size and enhancing lung deposition efficiency.

### 3.5. In Silico Aerodynamic Analysis

**Figure 5:**
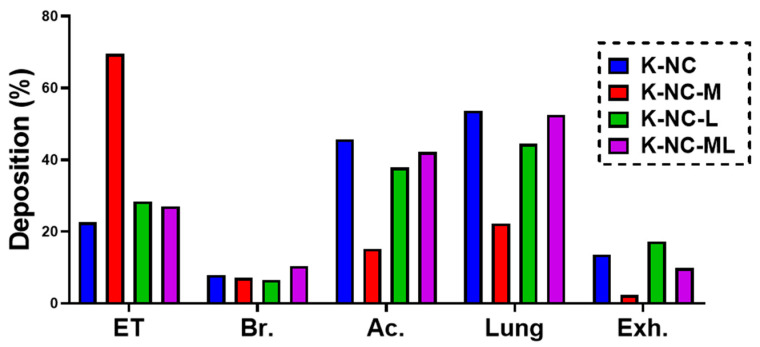
In silico results of deposited mass of NC-DP samples. ET: extra-thoracic, Br: bronchial, Ac: acinar, and EXH: exhaled.

For pulmonary delivery, the in silico model results largely aligned with findings from in vivo studies [63]. In this study, the model was employed to gain deeper insights into the aerosol performance of the samples in the airways. Aerodynamic analysis was conducted in silico with a 5-s breath-hold (BH) time, as shown in Figure 5.

The impact of excipients on aerosol behavior was evident in both the in silico and APS results, with general agreement between the two methods, except for notable differences in K-NC-L. However, as reported in the literature, in silico models often predict lower lung deposition than in vitro methods such as APS [64]. This discrepancy may stem from the simplified assumptions in computational models or the limitations of in vitro methods in replicating the complex dynamics of the human respiratory tract.

The inclusion of mannitol in K-NC-M increased the particle size and broadened the size distribution, leading to reduced lung deposition and higher extra-thoracic deposition, consistent with in vitro trends. However, K-NC-M also had the lowest exhaled fraction among all samples, likely due to its larger particle size reducing the likelihood of exhalation. In contrast, K-NC-L, which exhibited the highest aerosol performance in vitro due to its small particle size and narrow distribution, showed a higher exhaled fraction in silico. This may be because its smaller particles are more prone to exhalation in the simulated respiratory tract, whereas the APS results reflect superior dispersibility under controlled in vitro conditions.

The high lung deposition fractions observed for K-NC-ML (mannitol + leucine) align with previous findings [61], emphasizing the importance of balancing particle size and dispersibility to optimize lung deposition. While in vitro aerodynamic assessments (e.g., APS results) provide valuable insights into particle size distribution and aerosol performance, they may not fully predict actual lung deposition. In contrast, the in silico model, which simulates real-time airflow and particle dynamics in the respiratory tract, offers a more realistic evaluation of lung deposition, reinforcing its value in assessing deep lung delivery.

Inhalation time plays a crucial role in determining aerodynamic characteristics. Here, the inhalation time was relatively short (2.04 s), which is half the duration typically used in impactor-based measurements [34], and less than the time set in the APS study. Consequently, lung deposition could potentially be enhanced with a longer inhalation time. Studies have demonstrated that increasing the inhalation time can lead to a two-fold increase in drug deposition in the deep lung [65,66].

**Figure 6:**
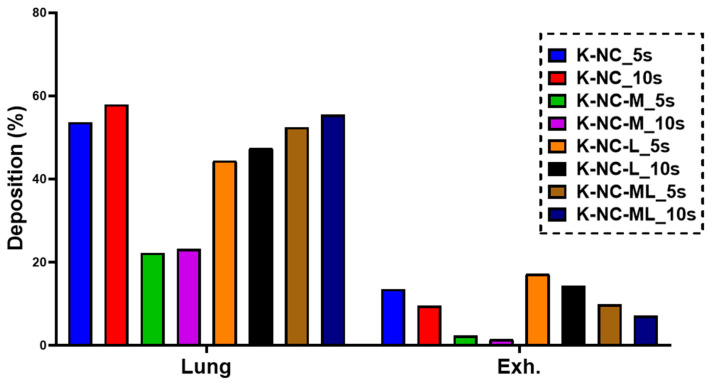
The effect of breath-holding time (5 and 10 s) on lung deposition and exhaled percentage examined by in silico modeling.

To further explore the impact of BH time on the lung deposition and exhaled fraction, an extended BH time (10 s) was evaluated. As shown in Figure 6, all formulations exhibited higher lung deposition with the prolonged BH time, while the exhaled fraction decreased. This emphasizes the significance of breath-holding in optimizing aerosol performance and maximizing drug delivery to the lungs.

### 3.6. Long-Term Stability Under Normal Conditions

To evaluate the stability of the four formulations (K-NC, K-NC-M, K-NC-L, and K-NC-ML), their particle size parameters (D[0.1], D[0.5], D[0.9]) and particle size distribution (span) were measured after six months of storage at room temperature in a desiccator (Table 7). The K-NC formulation, which lacked a stabilizing excipient, exhibited extreme particle growth, with D[0.9] increasing by over 6000% and span by nearly 6500%. This significant aggregation highlights the absence of stabilizers as a major factor contributing to instability [67]. In contrast, K-NC-M demonstrated moderate stability, with a 20% increase in D[0.9] and a 17% rise in span. These changes suggest that while mannitol provided some protective effects, it was not entirely effective in preventing aggregation. On the other hand, K-NC-L showed remarkable stability, with only a 1% increase in D[0.9] and a 5% increase in span. This suggests that leucine effectively minimized particle cohesion, preventing excessive aggregation [60].

The K-NC-ML formulation, which combined mannitol and leucine, showed a 6% reduction in D[0.5] and a 5% decrease in span. This improvement indicates enhanced dispersibility and stability, likely due to the synergistic effect of both excipients. Overall, these findings suggest that leucine, either alone or in combination with mannitol, plays a crucial role in maintaining particle size stability [31].

Taking into account the in silico results, the combination of mannitol and leucine in K-NC-ML exhibited enhanced aerosol performance alongside outstanding stability, compared to the excipient-free sample (K-NC) or single-excipient samples (K-NC-M and K-NC-L). This result aligns with findings from a previously published study, which reported that co-spray drying with sieved mannitol significantly enhanced the delivery efficiency of micronized budesonide compared to co-spray drying with l-leucine alone [68].

Given its strong performance, K-NC-ML was selected for further stability studies under stress conditions to assess its robustness for long-term storage and aerosol performance in dry powder inhalers.

### 3.7. Stability Assessment Under Accelerated Conditions

#### 3.7.1. Morphology

**Figure 7:**
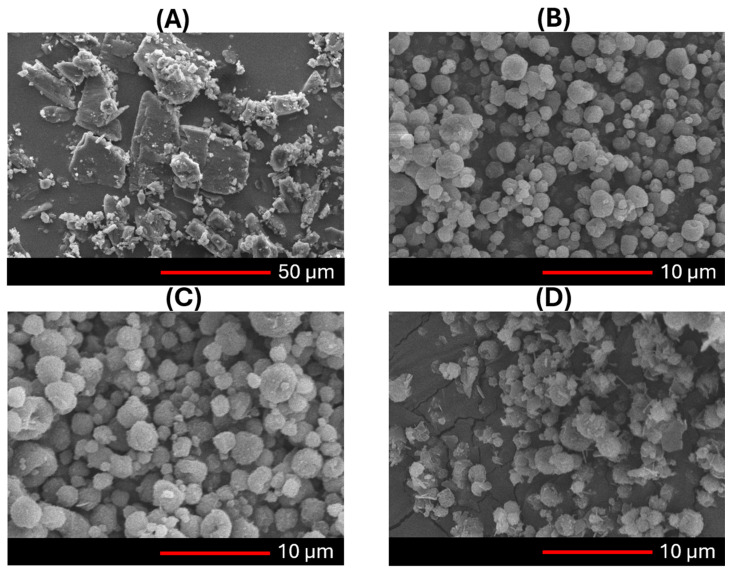
SEM images of (**A**) unprocessed KTP, and K-NC-ML sample at (**B**) 0 day, (**C**) 30 days, and (**D**) 90 days of storage under accelerated conditions (40 ± 2 °C and 75 ± 5% RH).

For dry powders intended for pulmonary delivery, maintaining specific size and shape characteristics is crucial to achieve effective deposition in the bronchioles or alveoli [69]. Particle morphology and size should be assessed collectively, as they are inherently interdependent [70]. SEM images of the NC-DP formulation (K-NC-ML) showed particles with an almost spherical morphology and a pollen-shaped surface (Figure 7). This near-spherical shape supports a uniform size distribution, while the rough surface enhances contact angles with lung tissues, improving aerodynamic performance. Particle shape significantly influences particle behavior. By carefully controlling shape, the flowability, aerosolization, and deposition performance of particles can be enhanced. Studies have shown that particles with a pollen-shaped surface demonstrate superior flowability, aerosolization, and deposition characteristics compared to other shapes [71]. The relationship between surface roughness and aerosolization arises from the increased number of contact points provided by a rougher surface [72]. Further examination of SEM images for K-NC-ML_3m revealed a slightly rougher surface, possibly resulting from solid-state changes during storage under high humidity [60]. Such changes might have caused uneven swelling or surface roughening over time [73]. Similar observations have been reported in a stability study involving leucine and various pharmaceutical actives, which confirmed that during storage, leucine-containing spray-dried powders can develop thin, fiber-like microstructures on their surface [32].

#### 3.7.2. Thermal and Solid-State Analysis

**Figure 8:**
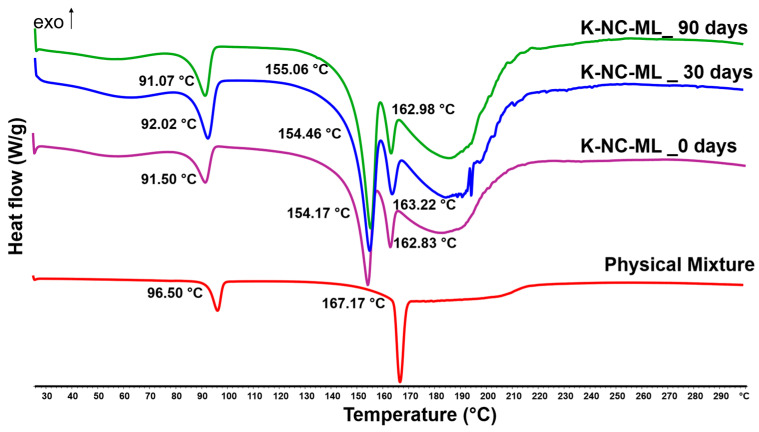
DSC thermal analysis of the physical mixture and sample K-NC-ML, analyzed as a freshly prepared sample (0 day) and after 30 and 90 days of storage under accelerated conditions (40 ± 2 °C and 75 ± 5% RH).

DSC was employed in this study to evaluate changes in the thermal stability and crystallinity of the samples during storage under stress conditions. Initially, the melting point of KTP in the physical mixture (PM) was observed at 96.50 °C (Figure 8). In the K-NC-ML sample, the melting point decreased to 91.50 °C, likely due to the milling process. Additionally, the broader peaks observed in K-NC-ML compared to PM suggested partial conversion of particles to an amorphous state after nano spray drying. Following storage, the endothermic peak’s position and intensity remained relatively stable, with melting points recorded at 92.02 °C after 30 days and 91.07 °C after 90 days. For mannitol, a single endothermic peak at 167.17 °C was noted in the PM, while two shifted peaks appeared at 154.17 °C and 162.83 °C after nano spray drying, likely indicating the presence of different polymorphs and distinct crystalline phases. Further investigations are required to verify the underlying mechanisms behind this phenomenon. A previously published article showed that the polymorphism of mannitol was affected after spray drying when added in different ratios with polystyrene beads [74]. Despite these changes, the peaks confirmed that mannitol remained in a crystalline form after nano spray drying. Hence, the DSC results demonstrated that the thermal stability of the samples remained largely unchanged throughout the storage period.

XRPD is a valuable technique for tracking structural modifications in spray-dried formulations over time, provided the diffraction patterns of the raw components are established. The physical state of the drug substance within the formulation plays a crucial role, as differences in crystallinity—whether amorphous or crystalline—can affect particle characteristics, interparticle forces, and ultimately, aerosol behavior.

**Figure 9:**
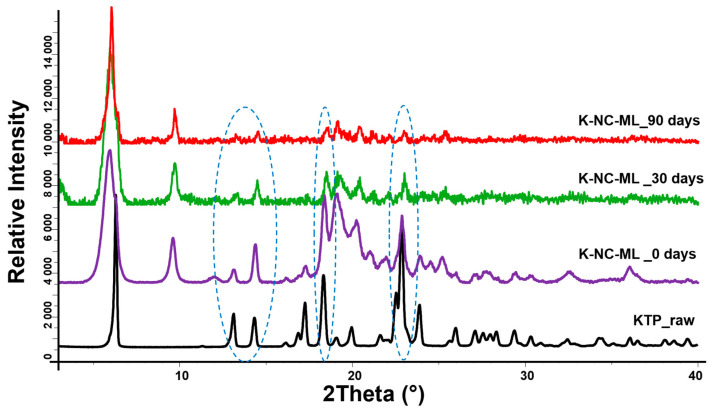
XRPD structural analysis of raw ketoprofen (KTP) and sample K-NC-ML, analyzed as a freshly prepared sample (0 days) and after 30 and 90 days of storage under accelerated conditions (40 ± 2 °C and 75 ± 5% RH). The circles highlight the characteristic peaks of KTP in the raw drug, as well as fresh and stored formulations.

The XRPD analysis (Figure 9) revealed distinct diffraction peaks for unprocessed KTP, with prominent reflections at 2θ angles of 13.5°, 14.53°, 17.23°, 18.48°, and 22.84°, confirming its crystalline nature. Notably, the K-NC-ML formulation maintained these key peaks post-production, demonstrating no loss of crystallinity. Additionally, these peaks persisted even after exposure to accelerated storage conditions. This observation is in line with previous studies, where formulations stored in HPMC capsules maintained consistent XRPD characteristic peaks under stress [75]. However, the rougher diffractograms with less intensity observed in the stored samples suggest a partial loss of crystalline order. This partial transition to an amorphous state could potentially influence powder performance, including aerodynamic characteristics and, consequently, therapeutic efficacy. Further investigations are needed to assess its impact in vivo.

#### 3.7.3. Aerodynamic Characterization by Andersen Cascade Impactor

The deposition profile of the K-NC-ML sample was assessed using the Andersen Cascade Impactor, with the aerodynamic performance analyzed via Inhalytix™ V 2.0 software. The freshly prepared K-NC-ML formulation showed a high fine particle fraction (FPF) of approximately 69.3%, outperforming commercial formulations designed for the Breezhaler^®^ device [76]. The emitted fraction (EF) was also notably high at ~93%, indicating efficient powder release from the inhaler.

**Figure 10:**
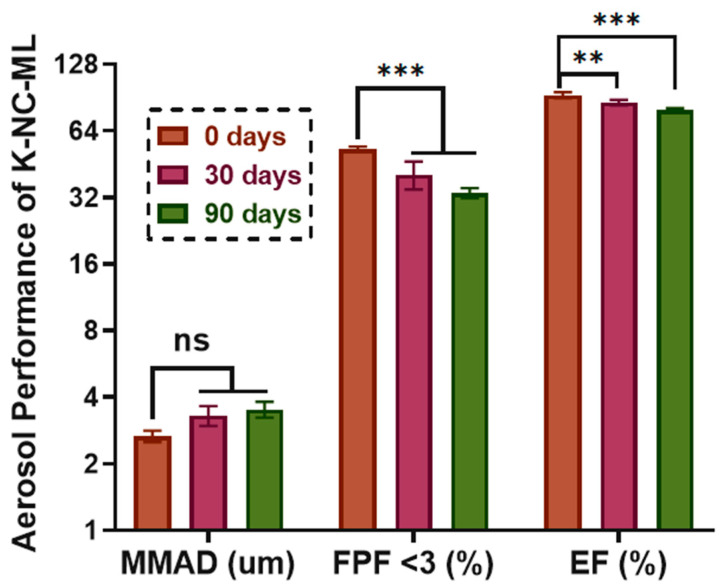
Significance of in vitro aerodynamic characteristics (MMAD, FPF, and EF) of the K-NC-ML sample, freshly prepared and after storage under accelerated conditions (40 ± 2 °C and 75 ± 5% RH), at a flow rate of 60 L/min. Results are presented as means ± SD (*n* = 3 independent measurements). Statistical significance is indicated as follows: ** *p* < 0.01, *** *p* < 0.001.

The aerodynamic performance is strongly influenced by particle size and morphology [29]. As discussed earlier, the inclusion of mannitol and leucine in the formulation played a key role in modifying particle properties. Their combined use as excipients significantly enhanced the aerodynamic characteristics of K-NC-ML. Notably, the mass median aerodynamic diameter (MMAD) remained stable during storage under stress conditions (Figure 10). However, a decline in FPF and EF values was observed, likely due to changes in surface morphology, such as increased roughness or partial particle aggregation caused by moisture absorption under stressed conditions and high RH.

Comparatively, the impact of RH on aerosolization behavior has been documented in previous studies. For instance, at 25 °C and 20% RH, the FPF of Inhalac 230 (lactose), salbutamol sulphate, and their binary mixtures increased by 19% compared to pre-storage levels, while at 25 °C and 43% RH, the FPF improved by 24% [77]. In contrast, Das et al. (2009) reported a significant reduction in the FPF of salmeterol xinafoate in a mixture containing 20% micronized lactose, decreasing from 11.3% to 7.7% at 75% RH, and further dropping to 4.9% at 95% RH [78].

**Figure 11:**
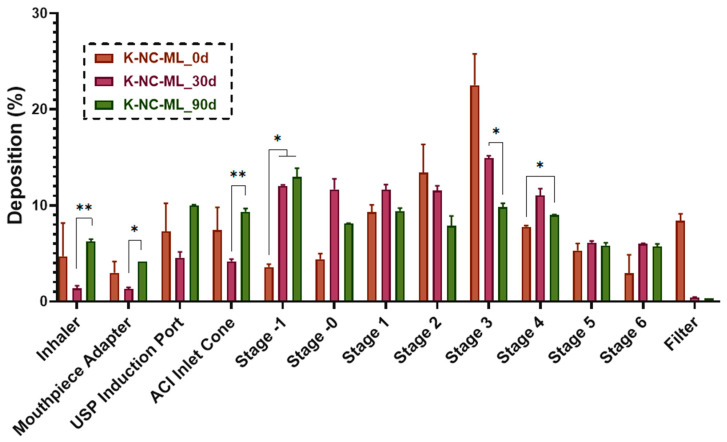
In vitro aerodynamic distribution of the K-NC-ML sample, freshly prepared and after storage under accelerated conditions (40 ± 2 °C and 75 ± 5% RH), at a flow rate of 60 L/min. Data are presented as means ± SD (*n* = 3 independent measurements). Statistical significance is indicated as follows: * *p* < 0.05, ** *p* < 0.01.

Figure 11 illustrates the particle distribution across impactor stages, highlighting effective KTP deposition from K-NC-ML before storage, particularly on stages one, two, and three, which correspond to deep respiratory regions [79]. Storage had no significant effect on deposition in stages one and two. Notably, no significant increase was observed in the USP induction port, confirming that the formulation maintained proper aerosol distribution even under stress conditions. Future studies should explore different capsule materials (e.g., gelatin capsules) and extended storage durations (e.g., six and twelve months) to further assess stability and aerosolization properties.

#### 3.7.4. Dissolution Test in Simulated Lung Media

**Figure 12:**
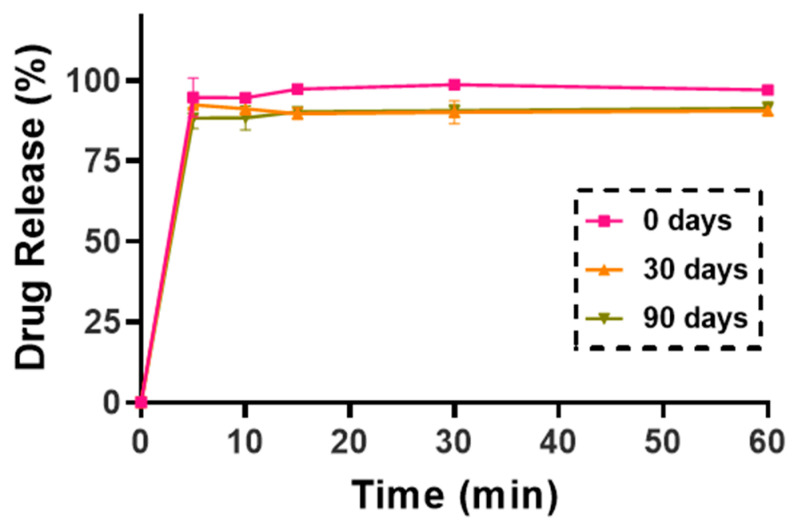
In vitro release study of physical mixture and nano spray-dried sample (K-NC-ML) as fresh and stored under accelerated conditions (40 ± 2 °C and 75 ± 5% RH), in simulated lung media (SLM). Results are expressed as mean ± SD (*n* = 3 independent measurements).

To mimic the lung environment, an in vitro dissolution test was performed using a simulated lung medium (pH 7.4). Particle engineering is widely recognized for producing fast-dissolving formulations [80]. In this study, the combined application of wet milling and nano spray drying as particle engineering techniques significantly increased the surface area, resulting in enhanced dissolution. As shown in Figure 12, sample K-NC-ML demonstrated rapid and extensive KTP release, achieving approximately 99% drug release within the first 5 min. This can be attributed to the nanocrystal form of the KTP produced, as the formation of nanocrystals greatly improved the dissolution rate of poorly water-soluble drugs [13]. Additionally, enhanced wettability prevented particle agglomeration during dissolution [81]. Mannitol, serving as a wettability enhancer and increasing the specific surface area, further promoted disintegration and accelerated drug release [82,83]. Notably, no significant difference was observed in the release profiles of the freshly prepared sample (at 0 days) and the stored samples (after 30 and 90 days of storage), suggesting that the formulation maintains consistent performance throughout its intended shelf life under normal storage conditions.

## 4. Conclusions

This study demonstrated the potential of nanocrystal-based dry powders (NC-DPs) for drug delivery via inhalation by assessing their aerodynamic performance and stability. The incorporation of mannitol and leucine, either individually or in combination, significantly influenced the particle size, stability, dispersibility, and deposition behavior. While leucine in K-NC-L improved dispersibility, it also led to a higher exhaled fraction in the in silico model. In contrast, mannitol (K-NC-M) reduced exhalation but was predominantly deposited in the extra-thoracic region. Notably, the combination of mannitol and leucine (K-NC-ML) achieved an optimal balance, exhibiting low exhaled fractions, high lung deposition, and superior long-term stability. Additionally, stability assessments confirmed that K-NC-ML maintained its crystallinity, thermal behavior, release profile, and aerodynamic particle size under accelerated conditions. Hence, the combination of mannitol and leucine in K-NC-ML provided a balance between stability and aerosol performance.

Overall, these findings highlight the importance of excipient selection in optimizing DPI formulations and reinforce the feasibility of NC_DP for enhancing pulmonary drug delivery. Future research should further explore alternative excipient types in spray drying and assess stability under various storage conditions, particularly at lower relative humidity levels.

## Figures and Tables

**Table 1:** Investigated parameters and levels.

Independent Factors	Factor Notation	Levels		
		−1	0	1
Pump (%)	X_1_	10	30	50
Temperature (°C)	X_2_	90	100	110
Feed concentration (% *w*/*v*)	X_3_	2.5	5	10

Pump (%) corresponds to mL/min.

**Table 2:** Samples annotation, composition, and concentration *.

Sample Name	KTP (% *w*/*v*)	Leucine (% *w*/*v*)	Mannitol (% *w*/*v*)
K-NC	5	0	0
K-NC-M	2.5	0	2.5
K-NC-L	2.5	2.5	0
K-NC-ML	2.5	1.25	1.25

* The concentrations of the samples were maintained to meet the optimized feed concentration of 5% *w*/*v*, as determined by the factorial design study.

**Table 3:** The particle sizes of NS as freshly prepared and after storage for 45 days according to the NTA.

Sample *	D[0.1] (nm)	D[0.5] (nm)	D[0.9] (nm)	Span
KTP-NS_0d	119.60 ± 3.30	168.70 ± 4.00	230.80 ± 11.70	0.659 ± 0.024
KTP-NS_45d	148.60 ± 1.80	193.40 ± 2.80	292.30 ± 5.80	0.743 ± 0.059

* KTP-NS-0d represents the fresh nanosuspension before storage, and KTP-NS_45d represents the nanosuspension after storage at 4 °C for 45 days.

**Table 4:** Experimental design of 15 runs with the independent variables and output responses.

Batch	Independent Variables			Dependent Variables		
	Pump (%)	Temp (°C)	Feed Conc. (%)	D[0.5] (µm)	Span	Yield (%)
1	10	90	5.0	1.9390	1.762	48.23
2	50	90	5.0	1.7080	1.412	92.54
3	10	110	5.0	2.2690	60.116	49.32
4	50	110	5.0	1.9320	1.967	64.23
5	10	100	2.5	1.9040	74.139	68.34
6	50	100	2.5	1.7550	17.948	65.80
7	10	100	10.0	2.3990	110.102	39.07
8	50	100	10.0	2.0995	1.634	61.69
9	30	90	2.5	2.0770	144.822	81.97
10	30	110	2.5	2.1170	183.908	71.27
11	30	90	10.0	2.0280	1.478	91.13
12	30	110	10.0	2.1800	4.375	53.52
13	30	100	5.0	1.9220	1.895	73.83
14	30	100	5.0	2.0795	11.789	63.27
15	30	100	5.0	2.0495	1.569	81.15

**Table 5:** Laser diffraction results of particle size represented by D[0.1], D[0.5], D[0.9], and span of NC-DP samples.

Sample	D[0.1] (µm)	D[0.5] (µm)	D[0.9] (µm)	Span
K-NC	0.863 ± 0.035	1.708 ± 0.074	3.271 ± 0.020	1.412 ± 0.071
K-NC-M	1.656 ± 0.185	3.337 ± 0.190	6.281 ± 0.051	1.390 ± 0.118
K-NC-L	0.993 ± 0.021	1.893 ± 0.017	3.691 ± 0.007	1.372 ± 0.070
K-NC-ML	1.072 ± 0.002	2.046 ± 0.064	3.910 ± 0.100	1.387 ± 0.008

Data are means ± SD (*n* = 3 independent measurements).

**Table 6:** The results of aerodynamic particle counting of NC-DP samples.

	Parameters	K-NC	K-NC-M	K-NC-L	K-NC-ML
Number particle size	Median (µm)	1.386 ± 0.019	1.690 ± 0.088	1.357 ± 0.028	1.600 ± 0.038
	Mean (µm)	1.510 ± 0.029	1.864 ± 0.113	1.469 ± 0.035	1.710 ± 0.037
	Geometric Mean (µm)	1.378 ± 0.021	1.666 ± 0.085	1.335 ± 0.027	1.550 ± 0.033
	Mode (µm)	1.382 ± 0.000	1.743 ± 0.104	1.444 ± 0.056	1.720 ± 0.000
	GSD *	1.530 ± 0.014	1.603 ± 0.033	1.546 ± 0.012	1.560 ± 0.009
Surface particle size	Median (µm)	3.673 ± 0.293	3.344 ± 0.547	2.394 ± 0.116	2.774 ± 0.130
	Mean (µm)	4.843 ± 0.267	4.424 ± 0.928	3.450 ± 0.369	4.098 ± 0.454
	Geometric Mean (µm)	4.045 ± 0.276	3.622 ± 0.637	2.692 ± 0.210	3.210 ± 0.249
	Mode (µm)	2.998 ±0.105	3.018 ± 0.295	2.358 ± 0.093	2.462 ± 0.124
	GSD *	1.800 ± 0.037	1.808 ± 0.126	1.880 ±0.084	1.900 ± 0.105
Volume particle size	Median (µm)	2.835 ± 0.200	2.610 ± 0.242	1.974 ± 0.065	2.262 ± 0.056
	Mean (µm)	3.445 ± 0.264	3.078 ± 0.433	2.280 ± 0.128	2.696 ± 0.139
	Geometric Mean (µm)	2.970 ± 0.244	2.662 ± 0.295	1.990 ± 0.083	2.330 ± 0.077
	Mode (µm)	2.690 ± 0.100	2.644 ± 0.134	2.040 ± 0.082	2.292 ± 0.117
	GSD *	1.688 ± 0.022	1.666 ± 0.085	1.620 ± 0.035	1.640 ± 0.049

* GSD: Geometric standard deviation. Data are means ± SD (*n* = 5 independent measurements).

**Table 7:** Laser diffraction results showing particle size parameters (D[0.1], D[0.5], D[0.9], and span) of samples after 6 months of storage, along with the percentage change relative to pre-storage values.

Sample	Storage	D[0.1] (µm)	D[0.5] (µm)	D[0.9] (µm)	Span
K-NC	6 months	0.960 ± 0.134	2.158 ± 0.095	201.87 ± 5.439	93.112 ± 7.821
	Change (%)	11	26	6072	6494
K-NC-M	6 months	1.738 ± 0.097	3.556 ± 0.289	7.511 ± 0.156	1.623 ± 0.312
	Change (%)	5	7	20	17
K-NC-L	6 months	0.985 ± 0.232	1.914 ± 0.193	3.731 ± 0.517	1.435 ± 0.089
	Change (%)	−1	1	1	5
K-NC-ML	6 months	1.036 ± 0.021	1.932 ± 0.057	3.595 ± 0.159	1.324 ± 0.074
	Change (%)	−3	−6	−8	−5

Data are means ± SD (*n* = 3 independent measurements).

## Data Availability

Data are contained within the article.
